# Cleavage and Polyadenylation Specificity Factor 6 Is Required for Efficient HIV-1 Latency Reversal

**DOI:** 10.1128/mBio.01098-21

**Published:** 2021-06-22

**Authors:** Yue Zheng, Heidi L. Schubert, Parmit K. Singh, Laura J. Martins, Alan N. Engelman, Iván D’Orso, Christopher P. Hill, Vicente Planelles

**Affiliations:** a Department of Pathology, School of Medicine, University of Utah, Salt Lake City, Utah, USA; b Department of Biochemistry, School of Medicine, University of Utah, Salt Lake City, Utah, USA; c Department of Cancer Immunology and Virology, Dana-Farber Cancer Institute, Boston, Massachusetts, USA; d Department of Medicine, Harvard Medical School, Boston, Massachusetts, USA; e Department of Microbiology, University of Texas Southwestern Medical Center, Dallas, Texas, USA; McMaster University

**Keywords:** HIV-1, CPSF6, PP2A, CDK9, Pol II, ITCH, transcription, latency, reactivation, proteasome

## Abstract

The HIV-1 latent reservoir is the major barrier to an HIV cure. Due to low levels or lack of transcriptional activity, HIV-1 latent proviruses *in vivo* are not easily detectable and cannot be targeted by either natural immune mechanisms or molecular therapies based on protein expression. To target the latent reservoir, further understanding of HIV-1 proviral transcription is required. In this study, we demonstrate a novel role for cleavage and polyadenylation specificity factor 6 (*CPSF6*) in HIV-1 transcription. We show that knockout of *CPSF6* hinders reactivation of latent HIV-1 proviruses by PMA in primary CD4^+^ cells. *CPSF6* knockout reduced HIV-1 transcription, concomitant with a drastic reduction in the phosphorylation levels of Pol II and CDK9. Knockout of *CPSF6* led to abnormal stabilization of protein phosphatase 2A (PP2A) subunit A, which then acted to dephosphorylate CDK9, downmodulating CDK9's ability to phosphorylate the Pol II carboxy-terminal domain. In agreement with this mechanism, incubation with the PP2A inhibitor, LB100, restored HIV-1 transcription in the CPSF6 knockout cells. Destabilization of PP2A subunit A occurs in the ubiquitin proteasome pathway, wherein CPSF6 acts as a substrate adaptor for the ITCH ubiquitin ligase. Our observations reveal a novel role of CPSF6 in HIV-1 transcription, which appears to be independent of its known roles in cleavage and polyadenylation and the targeting of preintegration complexes to the chromatin for viral DNA integration.

## INTRODUCTION

The major barrier to the eradication of HIV-1 infection is the presence of a small reservoir of latently infected cells that escape immune-mediated clearance (1–3). Due to the lack of transcriptional activity, latent proviruses *in vivo* are not easily detectable and cannot be targeted by either natural immune mechanisms or molecular therapies. Therefore, understanding how HIV-1 transcription is regulated will open doors to novel therapeutic strategies targeting the latent reservoir.

The positive transcription elongation factor (P-TEFb) is an essential host factor for HIV-1 gene expression (4). P-TEFb is a multiprotein complex containing the cyclin-dependent kinase CDK9 and a cyclin subunit, T1 or T2 (5). During gene transcription, active P-TEFb is recruited to the RNA polymerase II (Pol II) pause site and triggers the switch of the Pol II complex from an initiation mode into an elongation mode by phosphorylating the C-terminal domain of Pol II, the 5′6-dichloro-1-β-d-ribofuranosyl-benzimidaole-sensitive factor (DSIF), and the negative elongation factor (NELF) (6). P-TEFb typically exists in association with the 7SK snRNP complex in which hexamethylene bis-acetamide-inducible protein (HEXIM) bound to 7SK RNA inhibits the kinase activity of P-TEFb. The form of P-TEFb composed of CDK9 and cyclin T1 is known to be essential for HIV-1 transcription (7). HIV-1 Tat binds to cyclin T1 and prompts P-TEFb recruitment to Pol II, thereby boosting HIV-1 transcription (4, 8).

Cleavage and polyadenylation specificity factor 6 (CPSF6) is a member of the serine/arginine (SR)-rich protein family and has been found to bind to the HIV-1 capsid (9, 10). The C-terminal RS domain of CPSF6, which is a binding platform for the β-karyopherin transportin 3 (TNPO3), constitutes the protein’s nuclear localization signal (NLS) (11, 12). Cellular depletion of TNPO3 or truncation of the RS domain mislocalized CPSF6 to the cytoplasm and potently restricted HIV-1 infection (9, 11, 13). A single substitution in HIV-1 CA, N74D, can bypass CPSF6 binding and relieve the inhibitory effects of TNPO3 depletion on HIV-1 infection (9, 13–15). During the normal course of HIV-1 infection, CPSF6 facilitates viral nuclear entry (16, 17) and the targeting of speckle-associated genomic DNA regions for integration (18, 19).

The cellular function of CPSF6 involves the formation of a heterotetrameric protein complex, known as cleavage factor Im (CFIm), with CPSF5 (20, 21). The CFIm complex is involved in the earliest events of pre-mRNA cleavage prior to the addition of the poly(A) tail and, thus, regulates the general process of gene transcription (20). It has also been reported that CPSF6 has a role in transcription during development, as mutations in CPSF6 can lead to an interruption of gene expression in embryos (22).

Based on CPSF6's participation in cellular transcription, here we undertook efforts to ascertain whether CPSF6 plays a significant role in HIV-1 transcription in the context of latent infection. To that end, we used CRISPR/Cas9 technology in primary T cells (23, 24) to deplete CPSF6 in cells that had been latently infected with HIV-1. This method uses a vesicular stomatitis virus glycoprotein (VSV-G)-pseudotyped HIV-1 encoding the secreted enzyme nanoluciferase (nLuc), which provides a sensitive and quantitative measure of transcriptional output. We found that optimal reactivation of latent viruses required the presence of CPSF6. CPSF6 indirectly participated in HIV-1 transcription by inducing the ubiquitination and proteasomal degradation of protein phosphatase 2A (PP2A) scaffold subunit (also called subunit A) by ITCH E3 ligase. Through this mechanism, CPSF6 maintains low PP2A levels under basal conditions. Under conditions of CPSF6 knockout (KO), PP2A becomes stabilized and dephosphorylates CDK9, which then is unable to trigger Pol II to switch from initiation into elongation. This novel role of CPSF6 in viral transcription is independent of its role in cleavage and polyadenylation.

## RESULTS

### *CPSF6* KO hinders HIV-1 reactivation in primary CD4^+^ T cells.

**FIG 1:**
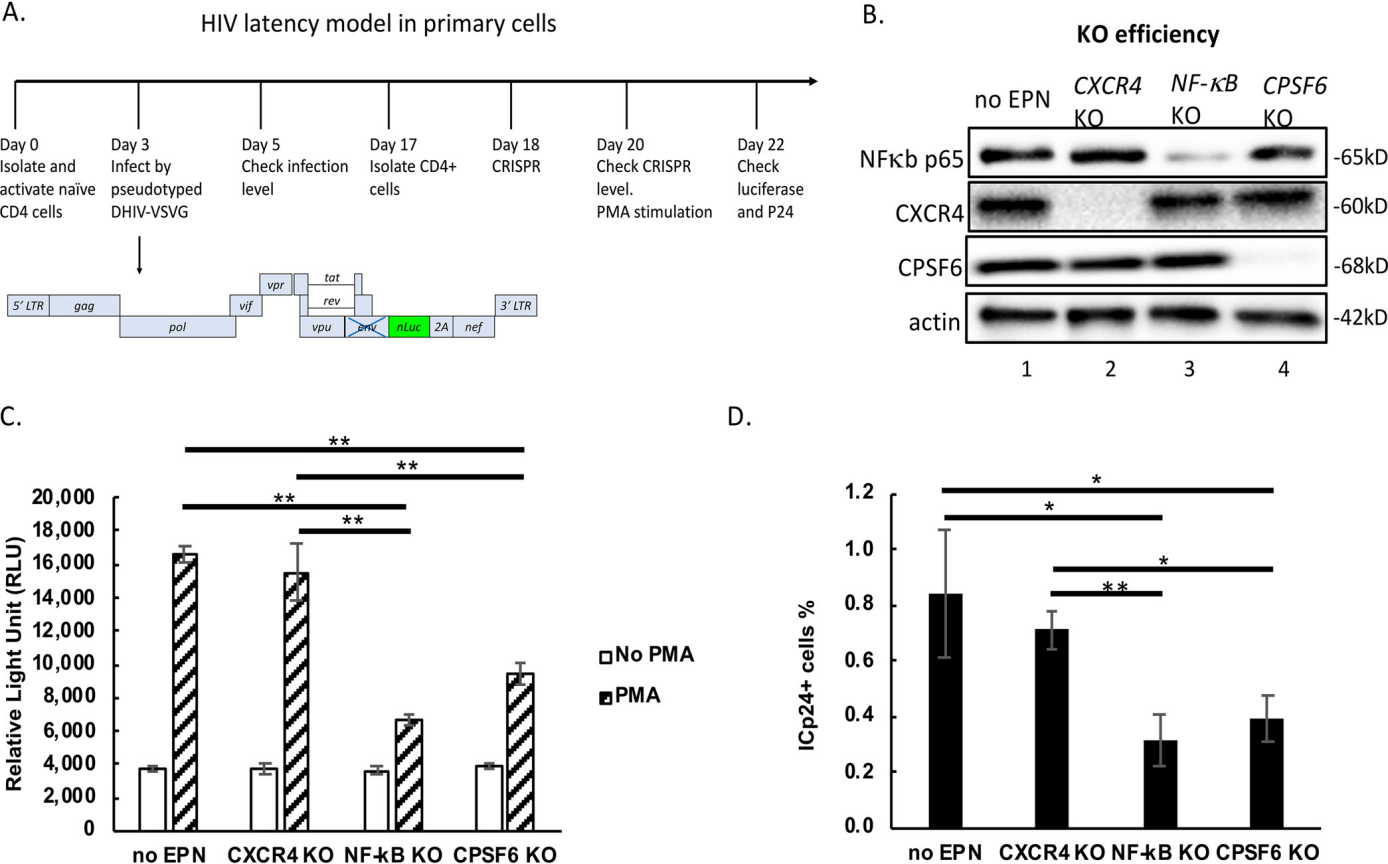
*CPSF6* KO hinders HIV-1 latency reversal by PMA in primary cells. (A) HIV-1 latency model in primary cells. (B) CRISPR/Cas9 RNP was delivered to HIV-1 latently infected primary cells (day 18 in panel A). KO efficiency was assessed by measuring expression of NF-κB p65, CXCR4, CPSF6, and actin via Western blotting 48 h thereafter and quantifying the band densitometry using the program Image Lab. (C) HIV-1 transcription was assessed by measuring nanoluciferase in the cell supernatants at day 22 in the presence or absence of PMA as indicated; no EPN, no electroporation. (D) Intracellular p24 expression was measured by flow cytometry at day 22; values shown represent percentages of p24^+^ cells in the PMA-treated sample minus those in untreated cells. Standard deviations (SD) represent the means from 3 experimental repeats. This experiment was performed three independent times. *P* values were calculated using Student's *t* test. *, *P* < 0.05; **, *P* < 0.01. Means ± SD from triplicates are shown.

To investigate the potential role of CPSF6 in HIV-1 latency, we used a primary cell model consisting of primary naive CD4^+^ T cells induced to differentiate into central memory T cells as previously described (25). For sensitive detection of transcriptional activity, we used a replication-defective virus encoding nLuc, which is secreted to the medium (Fig. 1A). In this experiment, we used the CRISPR ribonucleoprotein (RNP) method (26) to target genes of interest, as previously shown (23, 24). We used the following as controls for this experiment: no electroporation (no EPN; Fig. 1B, lane 1) and *CXCR4* KO (lane 2), as a nonrelevant gene KO. The KO efficiency for CXCR4 as assessed by immunoblotting cell lysates was 98%. We also targeted the NF-κB p65 subunit (71% efficiency; lane 3) and CPSF6 (81%; lane 4). Figure 1C shows the impact of the various KO treatments on the luciferase activities measured in the cell supernatants in the absence or in the presence of stimulation via phorbol 12-myristate 13-acetate (PMA), a potent protein kinase C (PKC) agonist. The no EPN control showed a 2.8-fold increase in response to PMA stimulation. In contrast, when NF-κB p65 was subjected to CRISPR/Cas9 KO, transcriptional activity in response to PMA achieved only a 1.7-fold increase, representing a 62% reduction in luciferase activity compared to the response of the no electroporation control to the PMA treatment. The reduction of luciferase activity by p65 KO confirmed the essential role of NF-κB in HIV-1 transcription under these conditions. Similar to p65 KO, KO of *CPSF6* significantly decreased luciferase production when stimulated by PMA (2-fold increase), representing a 47% reduction in luciferase activity compared to the response to the PMA-treated no electroporation control. Therefore, the effect of CPSF6 depletion was similar to that after depletion of NF-κB. In addition to PMA, we have also used anti-CD3/CD28 beads to reactivate HIV-1 latently infected cells. Depletion of CPSF6 diminished HIV-1 reactivation by stimulation with anti-CD3/CD28 antibodies as well, although to a lesser degree than in the case of PMA stimulation (see Fig. S1 in the supplemental material).

10.1128/mBio.01098-21.1 FIG S1 CPSF6 KO diminishes reactivation in response to both PMA and anti-CD3/CD28. HIV-1 latency experiment was performed as described for Fig. 1A. HIV-1 latently infected cells or uninfected cells were incubated with PMA or anti-CD3/CD28 beads for 48 h. Intracellular p24^+^ (ICp24^+^) cells were quantitated by flow cytometry at day 22. Download FIG S1, TIF file, 0.3 MB. Copyright © 2021 Zheng et al. 2021 Zheng et al. https://creativecommons.org/licenses/by/4.0/ This content is distributed under the terms of the Creative Commons Attribution 4.0 International license.

The experimental system described above also allows for measuring viral reactivation via expression of intracellular p24 (ICp24) protein in infected cells, measured by flow cytometry (Fig. 1D). The results closely parallel those obtained via luciferase activity, in that both p65 KO and *CPSF6* KO, but not *CXCR4* KO, hindered HIV-1 reactivation by PMA. These observations indicate that CPSF6 is required for optimal HIV-1 latency reversal by PMA. It is important to emphasize that in our experimental system, *CPSF6* KO is performed 15 days after infection with HIV-1, when most of the infectious events have already resulted in integration. Therefore, the emerging role of CPSF6 in proviral transcription appears to be independent of its previously reported role in nuclear targeting.

**FIG 2:**
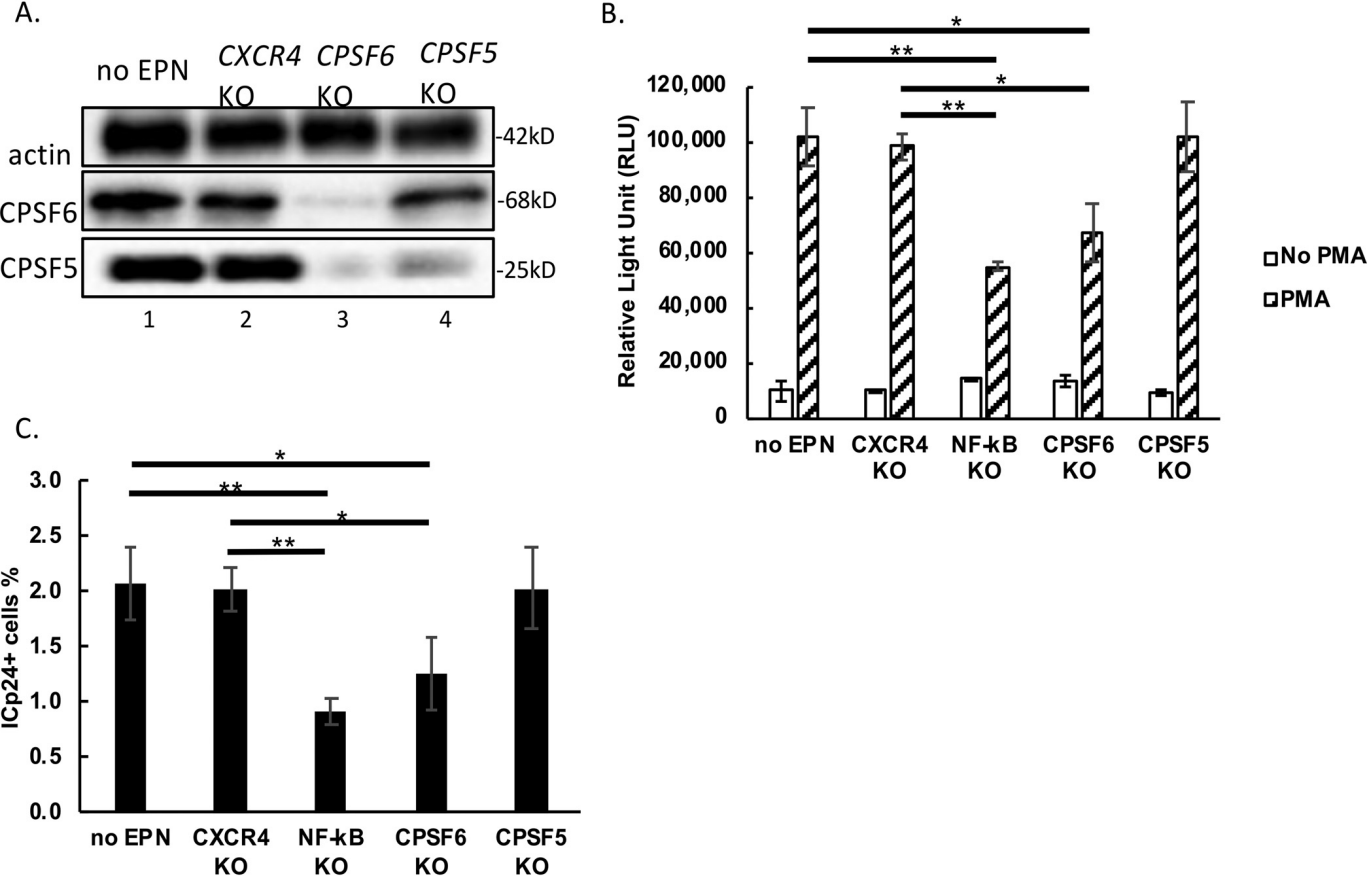
*CPSF5* KO does not hinder HIV-1 latency reversal by PMA. (A) CRISPR/Cas9 RNP was delivered to HIV-1 latently infected primary cells at day 18. CPSF5 KO and CPSF6 KO efficiency was assessed via expression of actin, CPSF5, and CPSF6 by Western blotting at 48 h after KO. (B) HIV-1 transcription was assessed by measuring nanoluciferase in the cell supernatants. (C) Intracellular p24 expression was measured by flow cytometry at day 22; values shown represent percentages of p24^+^ cells in the PMA-treated sample minus those in untreated cells. Standard deviations represent the means from 3 experimental repeats. This experiment was performed three independent times. *P* values were calculated using Student's *t* test. *, *P* < 0.05; **, *P* < 0.01.

Because CPSF6 is a component of the CFIm complex (20), we wished to examine whether CPSF6’s requirement in HIV-1 latency reversal was related to its function as part of the CFIm complex. To that end, we performed KO of *CPSF5*, which is CPSF6’s binding partner in the CFIm complex. As shown in Fig. 2A, CRISPR/Cas9-mediated KO of *CPSF6* also reduced CPSF5 protein levels, while KO of *CPSF5* did not change CPSF6 protein levels. Importantly, *CPSF5* KO had no discernible impact on HIV-1 latency reversal by PMA stimulation (Fig. 2B and C), suggesting that CPSF6’s role in polyadenylation is independent from its role in HIV-1 latency reversal.

### KO of *CPSF6* reduces HIV-1 gene transcription by inhibiting Pol II and CDK9 phosphorylation.

**FIG 3:**
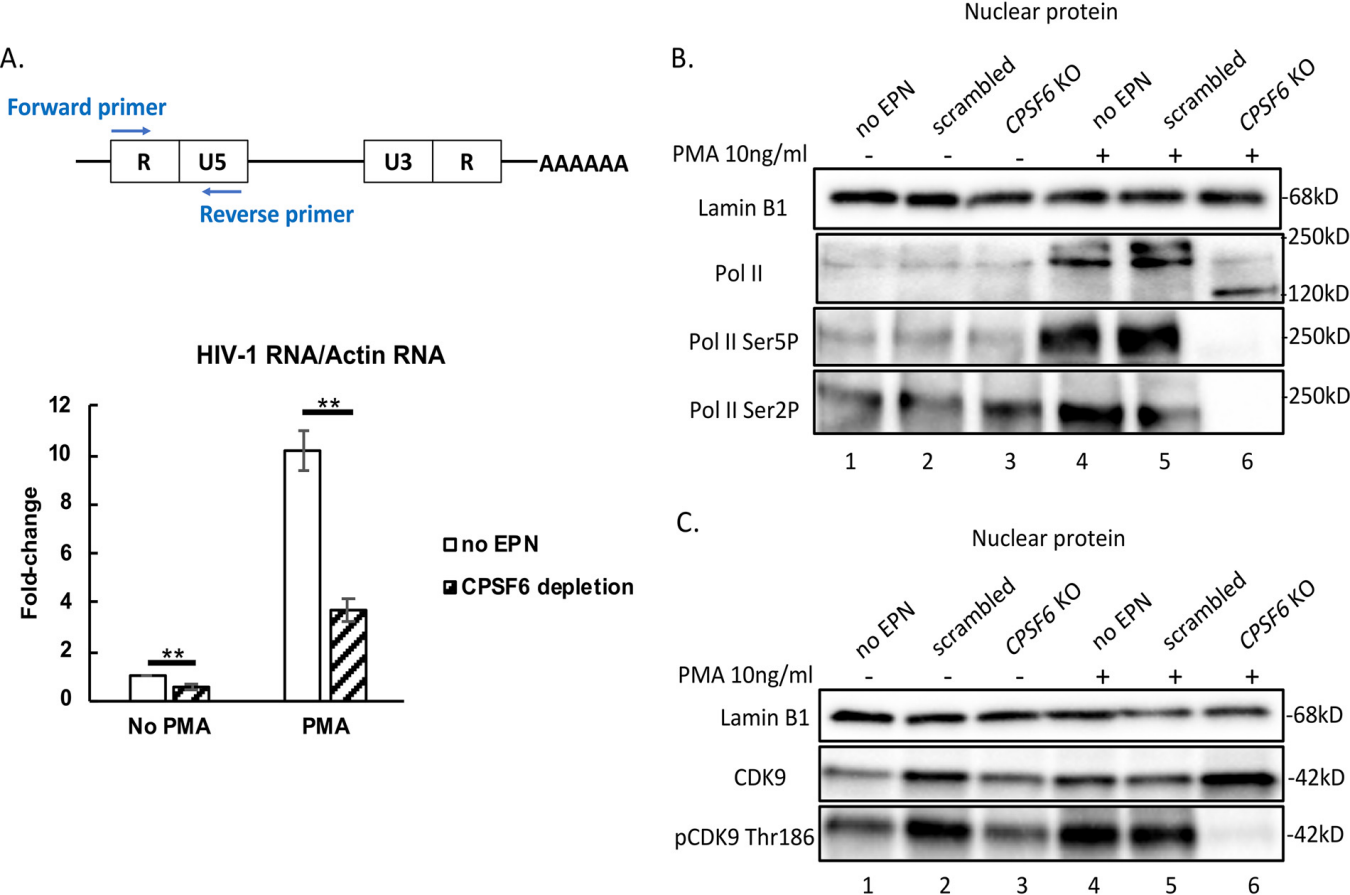
*CPSF6* KO reduces HIV-1 gene expression concomitant with dephosphorylation of Pol II and CDK9. (A) HIV-1 LTR-driven transcription was assessed by qPCR detecting a transcript between U5 and R (day 22). PCR quantitations were first normalized to *ACTB* (actin) and then to the no EPN control, which was assigned a value of 1. (B and C) Nuclear protein levels of indicated proteins, in the presence or absence of PMA, were detected by Western blotting at day 22. Standard deviations represent the means from 3 experimental repeats. This experiment was performed three independent times. Means ± SD from triplicates are shown. *P* values were calculated using Student's *t* test. *, *P* < 0.05; **, *P* < 0.01.

To further understand how CPSF6 participates in HIV-1 latency reversal, we assessed HIV-1-long terminal repeat (LTR)-driven gene expression by quantitative reverse transcription-PCR (qPCR) and used actin RNA for normalizing (Fig. 3A). As shown in Fig. 3A, compared to the no electroporation control, *CPSF6* KO decreased HIV-1-LTR-driven RNA expression by 40%. *CPSF6* KO, moreover, reduced LTR-driven transcription both in the absence and in the presence of cell stimulation (PMA).

RNA Pol II, which is the key enzyme catalyzing mRNA transcription, contains 52 heptad repeats of the sequence YSPTSPS in the carboxyl-terminal domain (CTD) of its largest subunit, RPB1. During the process of gene transcription, Pol II CTD is phosphorylated and dephosphorylated dynamically at different amino acid residues (serine, tyrosine, and threonine) within the heptad repeats. To examine whether CPSF6 loss can influence Pol II CTD phosphorylation in response to PMA stimulation, we measured both total Pol II and phosphorylated Pol II protein levels in the nucleus. In the absence of PMA stimulation, neither total nor phosphorylated Pol II was changed by KO of *CPSF6* (Fig. 3B, compare lanes 2 and 3). However, under PMA stimulation, KO of *CPSF6* was associated with a dramatic reduction of the phosphorylation levels of Pol II at Ser2 and Ser5 (Fig. 3B, compare lanes 5 and 6). The observed dephosphorylation of Pol II CTD is in agreement with a decrease in transcriptional output under conditions of *CPSF6* KO.

During HIV-1 transcription, P-TEFb is recruited by HIV-1 Tat to the Pol II pause site within the HIV-1 promoter. After becoming phosphorylated by the catalytic component of P-TEFb, phosphorylated CDK9 (p-CDK9), Pol II is released from the transcriptional pause to enter the elongation phase. To test whether CDK9 was responsible for the low phosphorylation levels of Pol II under conditions of *CPSF6* KO, we examined the total levels of CDK9 and phosphorylated CDK9 in the nucleus. We observed that the phosphorylation of CDK9 at Thr186 was markedly reduced under conditions of *CPSF6* KO in the presence of PMA (Fig. 3C, compare lanes 2, 3, 5, and 6).

### KO of *CPSF6* increases phosphatase PP2A protein levels.

**FIG 4:**
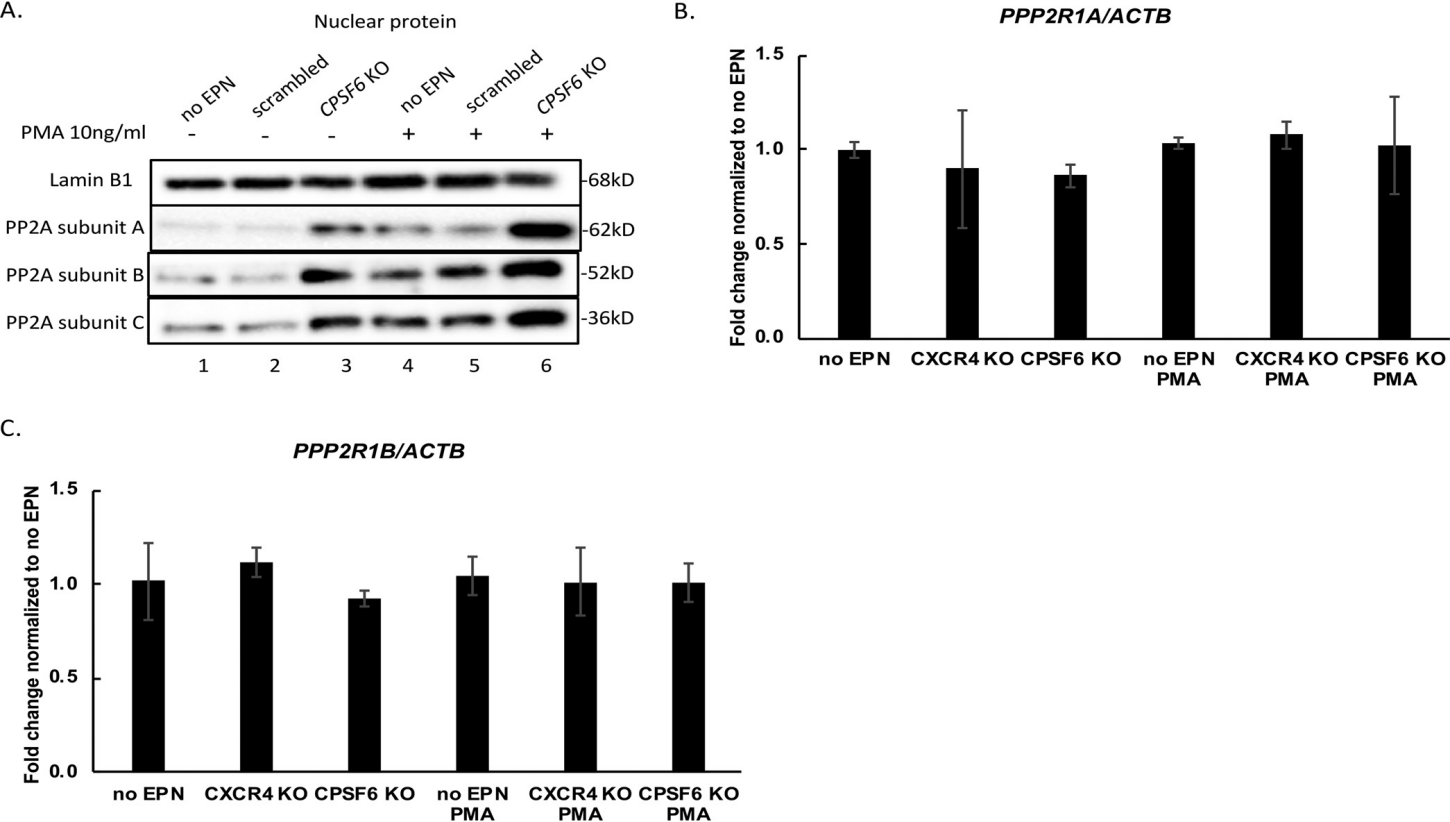
*CPSF6* KO is associated with dephosphorylation of CDK9 through increasing PP2A protein, but not mRNA, levels. (A) Nuclear protein levels of PP2A subunits A, B, and C were measured by Western blotting at day 22. (B and C) Gene expression levels of *PPP2R1A* and *PPP2R1B*, encoding PP2A subunit A isoforms Aα and Aβ, respectively, were measured via quantitative PCR. Data (means ± SD from triplicates) was normalized to *ACTB* (actin) and then to the no EPN control without PMA, which was set to 1. This experiment was performed 3 independent times.

It is known that PP2A can dephosphorylate CDK9 (27) and Pol II (28). Therefore, one potential explanation for the dephosphorylation of Thr186 of CDK9 and Pol II CTD is an increase in the level of PP2A expression. By immunoblotting, we indeed observed significant increases in PP2A subunit A, B, and C expression under *CPSF6* KO conditions irrespective of PMA (Fig. 4A, lanes 3 and 6). PP2A subunit A constitutes the scaffold for the PP2A complex, and conditions that compromise the stability of subunit A in turn destabilize subunits B and C (29). We measured the mRNA levels for the two known isoforms of subunit A, Aα and Aβ, encoded by *PPP2R1A* and *PPP2R1B*, respectively. There was no significant reduction of either *PPP2R1A* and *PPP2R1B* expression in *CPSF6* KO samples compared to samples in the no electroporation control or *CXCR4* KO in the presence or absence of PMA treatment (Fig. 4B and C), suggesting that the observed changes in protein levels were due to altered protein stability.

### The negative effect of *CPSF6* KO on HIV-1 transcription is reversed by PP2A inhibition.

**FIG 5:**
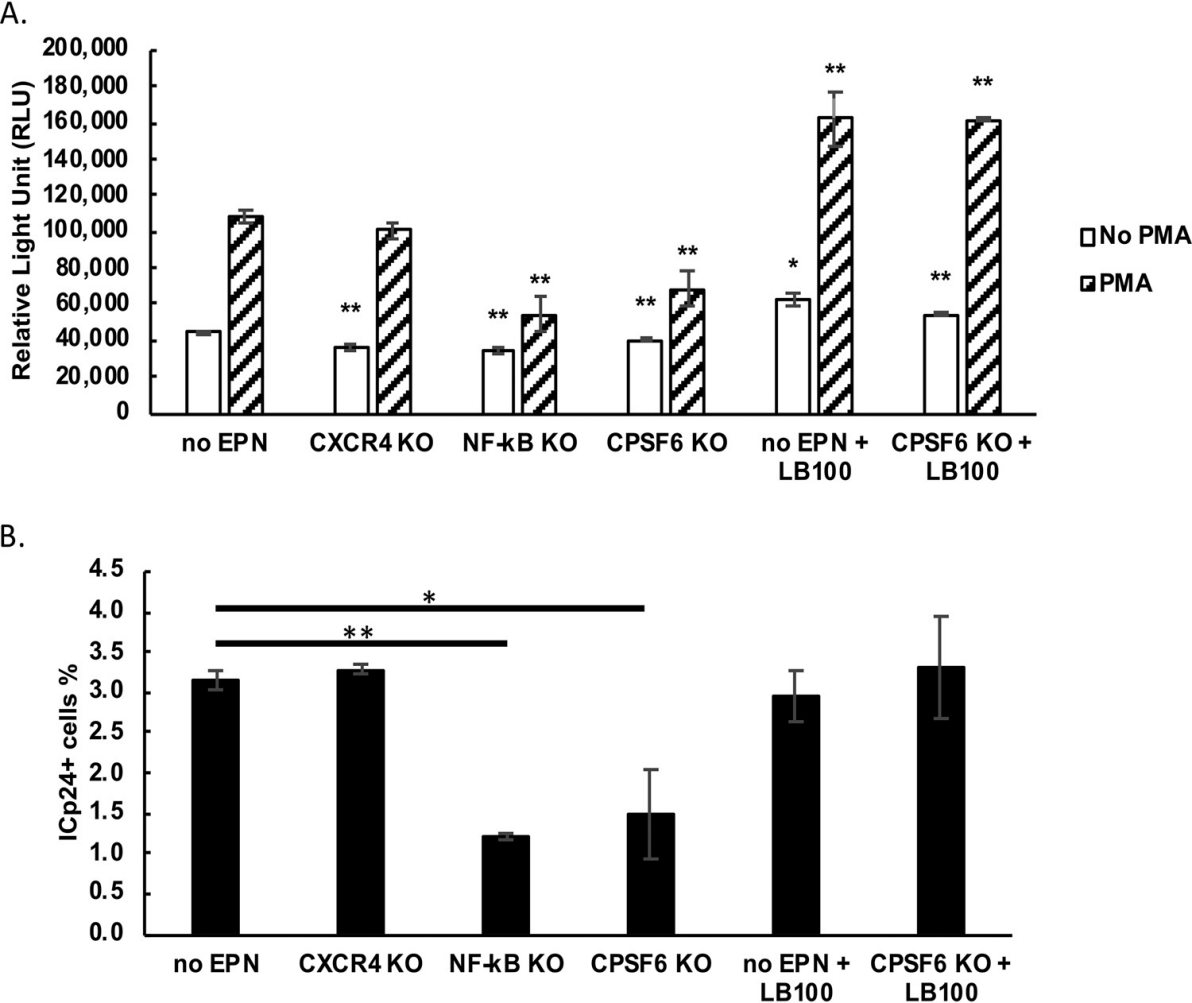
Inhibition of PP2A restores HIV-1 transcriptional activity under conditions of *CPSF6* KO. (A) HIV-1 transcription was assessed via nanoluciferase in cell supernatants in the presence or absence of the PP2A inhibitor, LB100, for 48 h (day 22). (B) HIV-1 intracellular p24 expression was measured in cells from the same samples as those shown in panel A at day 22; values shown represent percentages of p24^+^ cells in the PMA-treated sample minus those in untreated cells. Three independent experiments were performed, and one is shown. Means ± SD from triplicates are shown. *P* values were calculated using Student's *t* test. *, *P* < 0.05; **, *P* < 0.01.

To further validate whether the effect of *CPSF6* KO on HIV-1 latency reversal by PMA is exerted through an increase in PP2A function, we treated cells with the PP2A inhibitor LB100 (30). Incubation of latently infected *CPSF6* KO cells with LB100 restored luciferase values to the levels observed with the no EPN control, revealing full reactivation of HIV-1 proviral gene expression (Fig. 5A). LB100 treatment also efficiently reversed the reduction in ICp24-positive cells after *CPSF6* KO in HIV-1 latently infected cells stimulated by PMA (Fig. 5B).

### CPSF6 binds to PP2A subunit A, regulating its stability through the E3 ubiquitin ligase ITCH.

**FIG 6:**
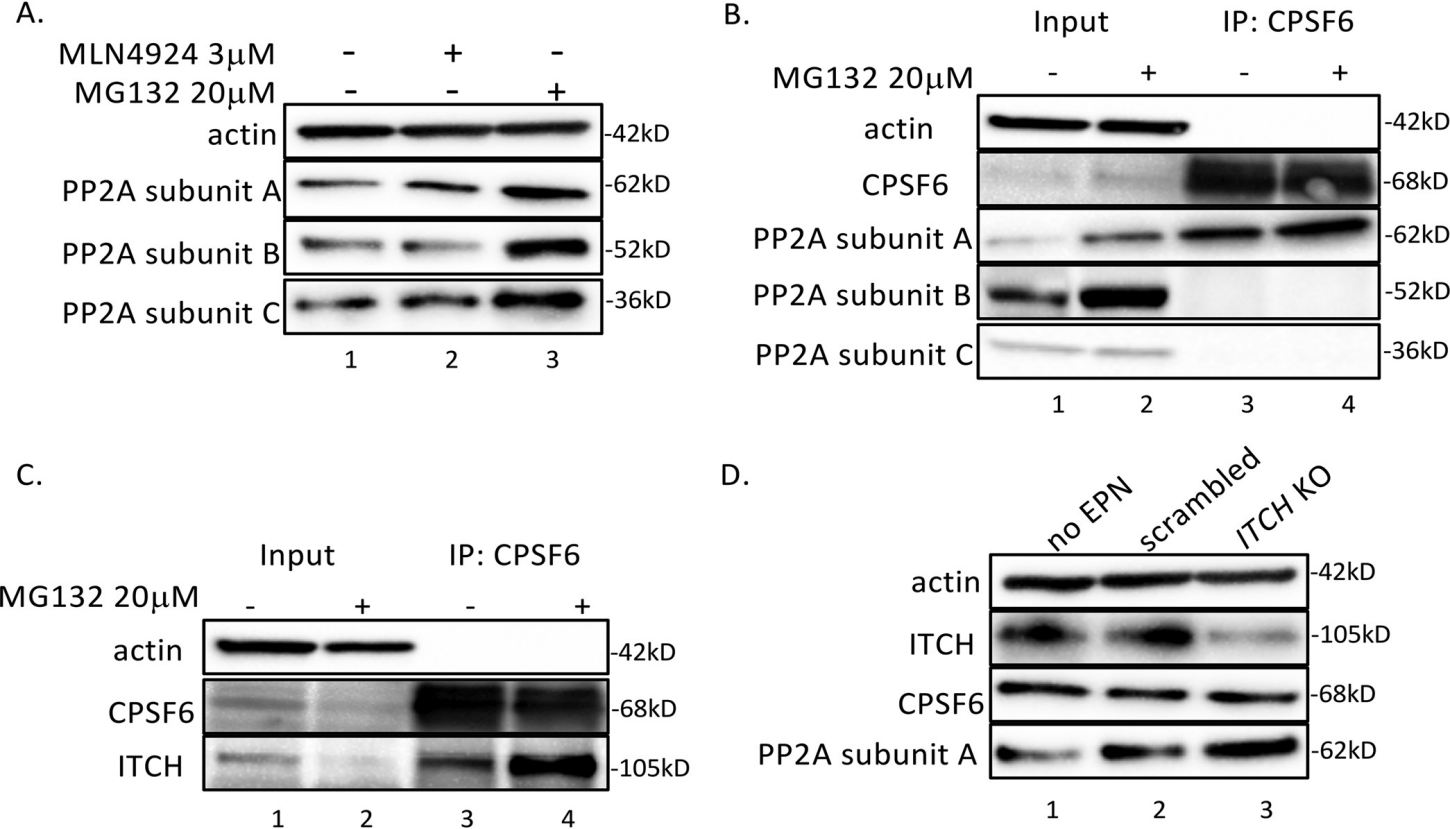
CPSF6 binds to PP2A subunit A and compromises its stability through the ubiquitin/proteasome pathway. (A) HEK293FT cells were treated with or without the proteasome inhibitor MG132 or the NEDD8-activating enzyme inhibitor MLN4924 for 24 h. Protein levels of PP2A subunits A, B, and C were measured by Western blotting. (B and C) CPSF6 and CPSF6-binding proteins were immunoprecipitated (IP) in HEK293FT cells treated with or without MG132. PP2A subunits A, B, and C and ITCH were detected by Western blotting. (D) ITCH was knocked out in SupT1 cells using CRISPR/Cas9 RNP for 2 days; protein levels of actin, ITCH, CPSF6, and PP2A subunit A were measured by Western blotting. This experiment was performed three independent times.

The stability and function of many proteins are regulated by the ubiquitin proteasome degradation pathway. To investigate how CPSF6 regulates PP2A protein levels, we first tested the proteasome inhibitor, MG132, and the neddylation inhibitor, MLN4924. Levels of PP2A subunit A, B, and C proteins increased after treatment with 20 μM MG132 but did not change after treatment with 3 μM MLN4924 (Fig. 6A) in HEK293FT cells. These data indicated that PP2A stability is controlled by the ubiquitin/proteasome degradation pathway but likely not by cullin E3 ligases, since their activity requires neddylation.

To determine if CPSF6 directly interacts with PP2A to regulate its stability through the protein degradation pathway, we performed coimmunoprecipitations of CPSF6 with PP2A subunits with or without MG132 treatment. As shown in Fig. 6B, PP2A subunit A, but not B or C, was coimmunoprecipitated with CPSF6 in HEK293FT cells. A previous study found CPSF6 as a potential binding partner of the ITCH E3 ligase (31). Based on this observation, we hypothesized that CPSF6 facilitates PP2A subunit A ubiquitination and degradation via ITCH. If this hypothesis were true, we would expect to find an interaction between CPSF6 and ITCH. Indeed, CPSF6 coprecipitated with ITCH (Fig. 6C). We have also confirmed the interaction of CPSF6, ITCH, and PP2A in primary CD4^+^ T cells via coimmunoprecipitation (Fig. S2).

10.1128/mBio.01098-21.2 FIG S2 ITCH and PP2A subunit A proteins were coimmunoprecipitated (IP) with CPSF6 in primary CD4^+^ T cells. Download FIG S2, TIF file, 0.6 MB. Copyright © 2021 Zheng et al. 2021 Zheng et al. https://creativecommons.org/licenses/by/4.0/ This content is distributed under the terms of the Creative Commons Attribution 4.0 International license.

Based on the potential role of ITCH in destabilizing PP2A subunit A, we predicted that KO of *ITCH* would lead to its stabilization. We probed PP2A protein levels in SupT1 cells in which ITCH was depleted via CRISPR/Cas9 technology. Interestingly, *ITCH* KO was associated with an increase in PP2A subunit A protein (Fig. 6D, compare PP2A subunit A in lane 2 versus 3). Therefore, KO of *ITCH* phenocopied *CPSF6* KO (Fig. 4A). *ITCH* KO had no effect on the levels of CPSF6 protein, in agreement with the idea that CPSF6 binds to ITCH as a substrate adaptor.

Overall, our data reveal that CPSF6 is required for optimal HIV-1 latency reversal by PMA. This effect is exerted through CPSF6’s ability to destabilize PP2A subunit A via the ubiquitin/proteasome pathway using ITCH as an E3 ligase.

## DISCUSSION

CPSF6 is known for its participation in two biological processes that fulfill cellular and viral roles. CPSF6 is a component of the cleavage factor Im (CFIm) (21), which regulates the cleavage and polyadenylation of mRNAs. The second known function of CPSF6 is to facilitate HIV-1 nuclear entry and to guide viral integration into transcriptionally active chromatin areas (16–19), with the participation of a plethora of other cellular factors, such as TNPO3, NUP153, NUP358, SUN1, SUN2, and cyclophilin A (reviewed in reference 32).

In the present study, we report that CPSF6 is required for optimal HIV-1 latency reversal and propose that this represents a third biological role for this critical nuclear protein. We also posit that the role of CPSF6 in transcription is independent of its previously known roles in alternative polyadenylation and preintegration complex targeting in HIV-1 infection.

Both CPSF5 and CPSF6 are required for the cleavage and polyadenylation process (33). CRISPR/Cas9 KO of *CPSF6* was associated with a reduction in viral reactivation efficiency in our experiments. However, KO of *CPSF5* had no appreciable effect on viral reactivation. Therefore, we conclude that the cleavage and polyadenylation function of CPSF6 is not required for CPSF6's role in transcriptional activation of HIV-1 following stimulation with PMA.

Likewise, the ability of CPSF6 to promote nuclear entry and chromosomal targeting of preintegration complexes also appears to be independent of the role in latent virus reactivation. In our experimental system, activated primary T cells are infected with a replication-defective virus and then allowed to return to a resting state for a period of 15 days. By this time, the early steps of the viral life cycle, which require the action of endogenous CPSF6, are completed. Therefore, KO of *CPSF6* at this time can only affect postintegration events, namely, the onset of transcription following stimulation with PMA.

Transcription is a multistep process mediated by RNA Pol II (reviewed in reference 34). RNA Pol II is recruited to the HIV-1 promoter to form a preinitiation complex along with general transcription factors (34, 35). Pol II synthesizes an initial transcript of about 20 to 60 nucleotides, and then it enters a pause mode concomitant with the binding of DSIF and NELF to the complex (36). HIV-1 efficiently overcomes the paused state of Pol II via recruitment of P-TEFb by Tat. P-TEFb then phosphorylates Pol II-CTD, DSIF, and NELF. These phosphorylation events lead to dissociation of NELF and the switch of Pol II from the pause mode to the elongation mode (4, 7, 37). A recent study has also shown that PP2A directly binds to Pol II and cooperates with the integrator complex to directly regulate the phosphorylation levels of Pol II CTD (28).

Our experiments showed that KO of *CPSF6* reduced the amounts of phosphorylated CDK9 (but not total CDK9) and phosphorylated Pol II as well as the levels of luciferase produced by the provirus. Therefore, CPSF6 is required for HIV-1 transcription elongation. Based on previous reports on the ability of PP2A to dephosphorylate CDK9 as well as Pol II CTD (27, 28, 38), we probed the potential role of PP2A in this process. Two pieces of evidence support the role of PP2A in transcription in general. First, PP2A regulatory subunit A was stabilized under conditions of *CPSF6* KO, suggesting that CPSF6 controls PP2A stability. Second, pharmacological inhibition of PP2A completely relieved the inhibitory effect of *CPSF6* KO.

We investigated the mechanism of PP2A destruction by incubating cells with the proteasome inhibitor MG132, which resulted in stabilization of PP2A. Based on the notion that CPSF6 was found in association with ITCH, a known E3 ubiquitin ligase (31, 39), we depleted ITCH via CRISPR/Cas9, and this resulted in stabilization of PP2A.

qPCR analysis of the mRNAs for the PP2A subunit A revealed no significant differences between the no electroporation control, *CXCR4* KO, and *CPSF6* KO samples, indicating that differences in gene expression did not account for the observed variations in PP2A protein levels.

We confirmed that CPSF6 can be immunoprecipitated with ITCH (31, 39). In addition, we found that CPSF6 could be immunoprecipitated with PP2A subunit A but not B or C. Destabilization of subunit A by the CPSF6/ITCH complex, therefore, compromises the stability of subunits B and C, as previously shown (29).

Based on our observations, we propose that CPSF6 is a substrate adaptor for ITCH. Other known substrate adaptors for ITCH are the Nedd4 family interacting protein-1 (NDFIP1) and Numb, which mediate degradation of phosphatase and tensin homolog (PTEN) and glioma-associated oncogene homolog 1 (Gli1), respectively (40, 41). Both NDFIP1 and Numb bind to ITCH's WW domain via their PY/PPXY motif. CPSF6 also encodes a PPXY motif, which, when mutated (Y to A), abolished the interaction between CPSF6 and ITCH (39).

**FIG 7:**
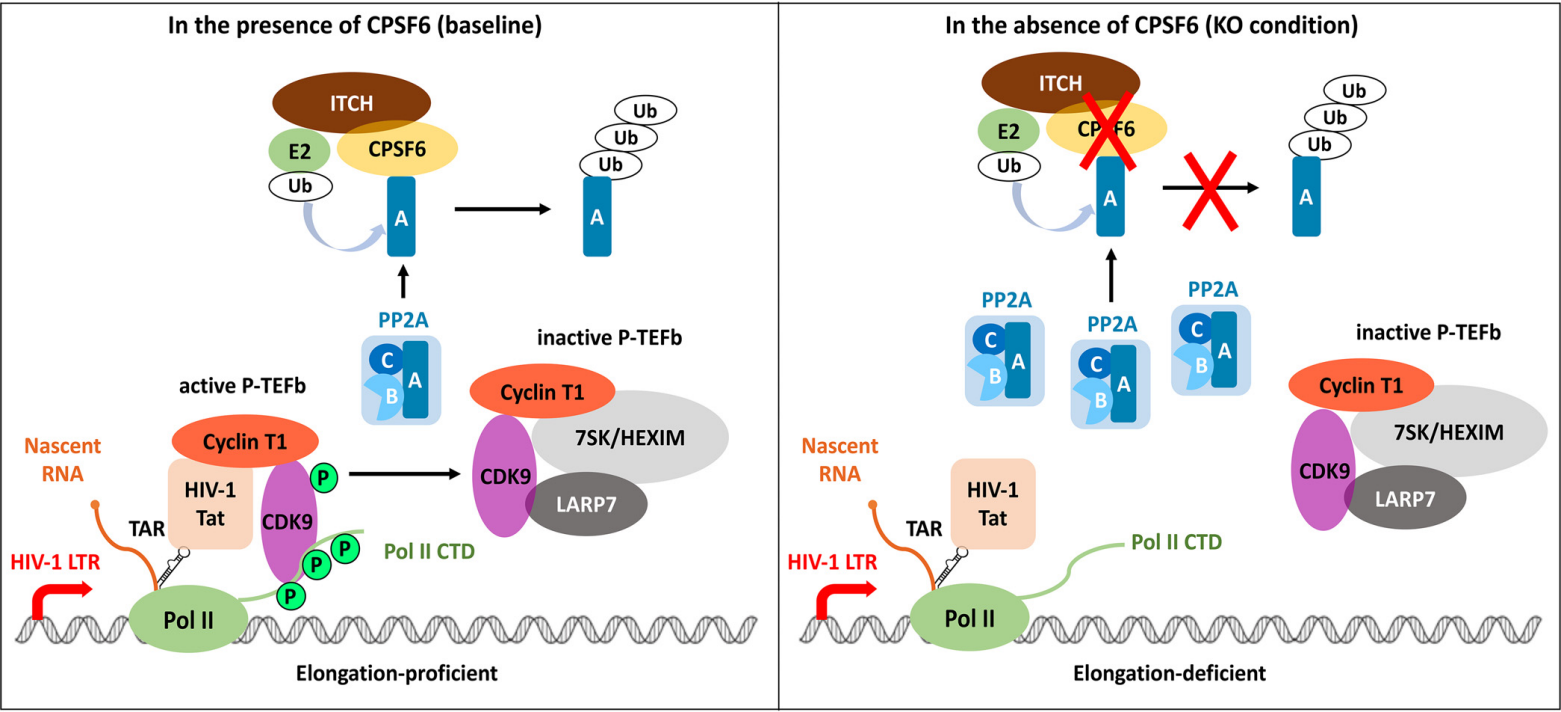
Model of CPSF6 regulating HIV-1 gene transcription through assisting PP2A degradation via ITCH E3 ligase. Under normal conditions (baseline), ITCH E3 ligase and CPSF6 maintain PP2A subunit A at low levels. HIV-1 Tat binds to cyclin T1 and recruits active P-TEFb (cyclin T1 and phosphorylated CDK9) to the TAR loop on the nascent RNA. Thereafter, P-TEFb phosphorylates the Pol II CTD, an event that releases the Pol II complex into an elongation mode. In the absence of CPSF6 (KO condition), PP2A subunit A is no longer ubiquitinated by the ITCH E3 ligase and becomes stabilized. The phosphate residues of CDK9 and Pol II CTD are removed. Under *CPSF6* KO condition, the Pol II complex fails to enter the elongation mode.

A model depicting our findings is shown in Fig. 7. In the presence of CPSF6 (baseline conditions), PP2A is destabilized and unable to dephosphorylate CDK9 residue Thr186, maintaining P-TEFb in an active form and stimulating transcription elongation from the HIV-1 promoter. Ectopic removal of CPSF6 results in aberrant stabilization of PP2A, which then efficiently removes the phosphate from Thr186 of CDK9, rendering P-TEFb inactive and unable to induce Pol II to switch to the elongation mode.

## MATERIALS AND METHODS

### Cell culture.

HEK293FT cells were purchased from the ATCC and cultured in Dulbecco’s modified Eagle’s medium (DMEM; Gibco) containing 10% fetal bovine serum (FBS; Gibco), 2 mM l-Glu (Gibco). SupT1 cells were obtained from the NIH AIDS Reagent Program and cultured in RPMI 1640 containing 10% FBS and 2 mM l-Glu.

### Plasmid DNAs.

Plasmid pCMV-VSVG was purchased from Addgene. pNL4.3-ΔEnv-nLuc-2ANef was previously described (24, 25).

### Virus production.

Pseudotyped viruses, pNL4.3-ΔEnv-nLuc-2ANef-VSVG (DHIV-VSVG), were produced by cotransfecting pNL4.3-ΔEnv-nLuc-2ANef and pCMV-VSVG into HEK293T cells using the calcium phosphate transfection method (see Text S1 in the supplemental material).

10.1128/mBio.01098-21.6 TEXT S1 Virus production. Download Text S1, DOCX file, 0.01 MB. Copyright © 2021 Zheng et al. 2021 Zheng et al. https://creativecommons.org/licenses/by/4.0/ This content is distributed under the terms of the Creative Commons Attribution 4.0 International license.

### CRISPR-Ca9-mediated KO.

Guide RNAs (gRNAs) (Table S1; IDT) were mixed with tracrRNA (IDT) and heated at 95°C for 5 min and then mixed with recombinant Streptococcus pyogenes Cas9 nuclease (IDT). Preassembled Cas9-gRNA RNPs were electroporated into cells using a Neon unit (ThermoFisher). After 2 days, knockout efficiency was measured via either flow cytometry or Western blotting (Text S2).

10.1128/mBio.01098-21.3 TABLE S1 Sequences of gRNAs. Download Table S1, DOCX file, 0.01 MB. Copyright © 2021 Zheng et al. 2021 Zheng et al. https://creativecommons.org/licenses/by/4.0/ This content is distributed under the terms of the Creative Commons Attribution 4.0 International license.

10.1128/mBio.01098-21.7 TEXT S2 CRISPR-Ca9-mediated KO. Download Text S2, DOCX file, 0.01 MB. Copyright © 2021 Zheng et al. 2021 Zheng et al. https://creativecommons.org/licenses/by/4.0/ This content is distributed under the terms of the Creative Commons Attribution 4.0 International license.

### Generation of HIV-1 latency model in primary cells.

The HIV-1 latency model in primary cells was adapted from a previous model (25). Briefly, peripheral blood mononuclear cells (PBMCs) were isolated by venipuncture from healthy, deidentified donors using Lymphoprep (STEMCELL Technologies) and cultured in complete medium (RPMI 1640 with 10% FBS and 2 mM l-Glu). Naïve cells were purified using the EasySep human naïve CD4^+^ T cell isolation kit (STEMCELL Technologies) and then activated by culturing in a 96-well plate with 1 μg/ml anti-interleukin-4 (IL-4) antibody (Peprotech), 2 μg/ml anti-IL-12 antibody (Peprotech), 10 ng/ml TGF-β1 (Peprotech), and anti-CD3/CD28 antibody beads (1 bead/cell) (Gibco). After 3 days, beads were removed via a magnetic column (STEMCELL Technologies). Cells were infected with pseudotyped pNL4.3-ΔEnv-nLuc-2ANef-VSVG viruses using the spinoculation method. Infected and uninfected cells were cultured in complete medium with 30 IU/ml IL-2 (NIH AIDS Reagent Program). At day 5, cells were stained with fixable viability dye eFluor 450, anti-CD4-allophycocyanin (APC), and anti-HIV-1 core (P24)-fluorescein isothiocyanate (FITC) and analyzed by flow cytometry in a BD LSRFortessa X-20 (Text S3). At day 17, CD4^+^ cells were isolated using a Dynabeads CD4-positive isolation kit (STEMCELL Technologies). At day 18, CD4^+^ cells were placed in a 96-well plate and treated with or without PMA (Sigma) or LB100 (MedKoo). After 2 days, luciferase values of cell supernatants were measured by the Nano-Glo luciferase assay system (Promega). Cells were stained with fixable viability dye eFluor 450, anti-CD4-APC, and anti-HIV-1 core (P24)-FITC and analyzed by BD LSRFortessa X-20. Data were analyzed via FlowJo 10.7.1.

10.1128/mBio.01098-21.8 TEXT S3 Flow cytometry. Download Text S3, DOCX file, 0.01 MB. Copyright © 2021 Zheng et al. 2021 Zheng et al. https://creativecommons.org/licenses/by/4.0/ This content is distributed under the terms of the Creative Commons Attribution 4.0 International license.

### RNA isolation and qPCR.

Total RNA was isolated from freshly collected cells using an RNeasy minikit (Qiagen). cDNA synthesis was done using the SuperScript IV first-strand synthesis system (Invitrogen). qPCR was performed using Platinum SYBR green qPCR SuperMix-UDG (Invitrogen) on a LightCycler 480 (Roche), and fold changes were calculated as described by the manufacturer. Oligonucleotides for *ACTB* were obtained from the SYBR green Cells-to-CT control kit, while others are listed in Table S2.

10.1128/mBio.01098-21.4 TABLE S2 Sequences of qPCR primers. Download Table S2, DOCX file, 0.01 MB. Copyright © 2021 Zheng et al. 2021 Zheng et al. https://creativecommons.org/licenses/by/4.0/ This content is distributed under the terms of the Creative Commons Attribution 4.0 International license.

### Western blotting.

Total protein and nuclear protein (Text S4) were electrophoresed on a precast 4 to 15% polyacrylamide SDS-PAGE gel (Bio-Rad) and transferred onto a 0.45-μm polyvinylidene difluoride (PVDF) membrane (Sigma). Membranes were then blocked in 3% bovine serum albumin–Tris-buffered saline with Tween 20 (BSA-TBST; Sigma) by shaking at room temperature for 1 h and incubated with primary antibodies (Table S3) with shaking at 4°C overnight. After 3 washes with TBST, membranes were incubated with secondary antibodies on a rocker at room temperature for 2 h. Pierce ECL Western blotting substrate (ThermoFisher) was used to develop immunoblots. Images were taken with a Bio-Rad Gel Doc (Bio-Rad). Analysis of band densitometry was done using Image Lab software (Bio-Rad).

10.1128/mBio.01098-21.5 TABLE S3 Antibodies for Western blotting. Download Table S3, DOCX file, 0.02 MB. Copyright © 2021 Zheng et al. 2021 Zheng et al. https://creativecommons.org/licenses/by/4.0/ This content is distributed under the terms of the Creative Commons Attribution 4.0 International license.

10.1128/mBio.01098-21.9 TEXT S4 Protein extracts for Western blotting. Download Text S4, DOCX file, 0.01 MB. Copyright © 2021 Zheng et al. 2021 Zheng et al. https://creativecommons.org/licenses/by/4.0/ This content is distributed under the terms of the Creative Commons Attribution 4.0 International license.

### Coimmunoprecipitation.

Dynabeads were precoated with rabbit anti-human CPSF6 (number 175237; Abcam) or rabbit IgG isotype control (number 172730; Abcam) at 4°C for 4 h (ThermoFisher). Cell lysates were added to antibody-precoated beads and kept rolling at 4°C overnight. Proteins were then eluted in NETN^+/+^-containing 1× Laemmli buffer (2% SDS, 0.1% bromophenol blue, 7.8% glycerol, 10 mM Tris, pH 6.8, 1.5% dithiothreitol) by heating at 95°C for 10 min. The proteins were further analyzed by Western blotting.
