# Supplementary figures and images for: Cleavage and Polyadenylation Specificity Factor 6 Is Required for Efficient HIV-1 Latency Reversal
Source: mBio. 2021 Jun 22;12(3):e01098-21. doi: 10.1128/mBio.01098-21 (PMC8262898; doi:10.1128/mBio.01098-21)

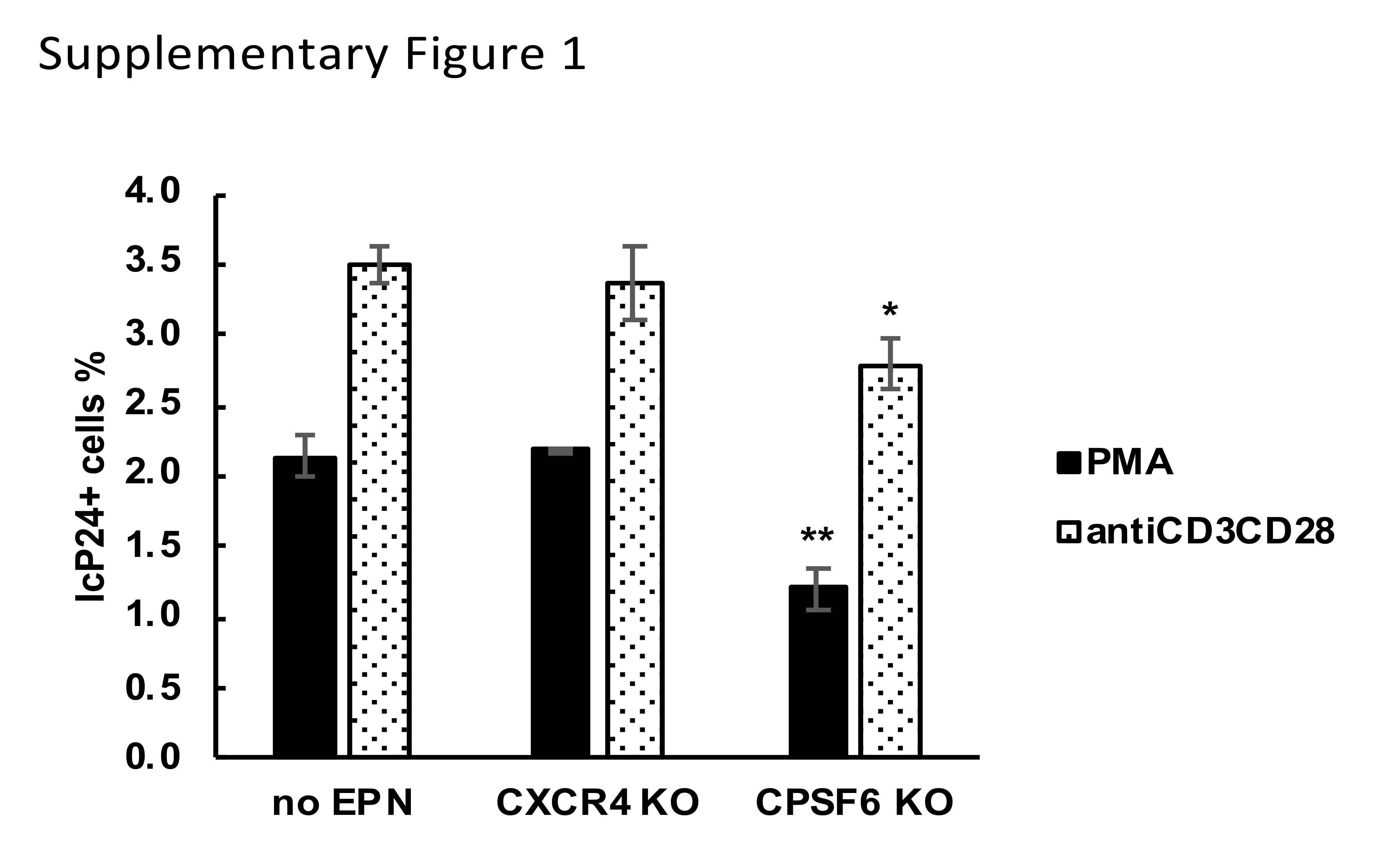

Supplement: FIG S1 [file mbio.01098-21-sf001.tif]

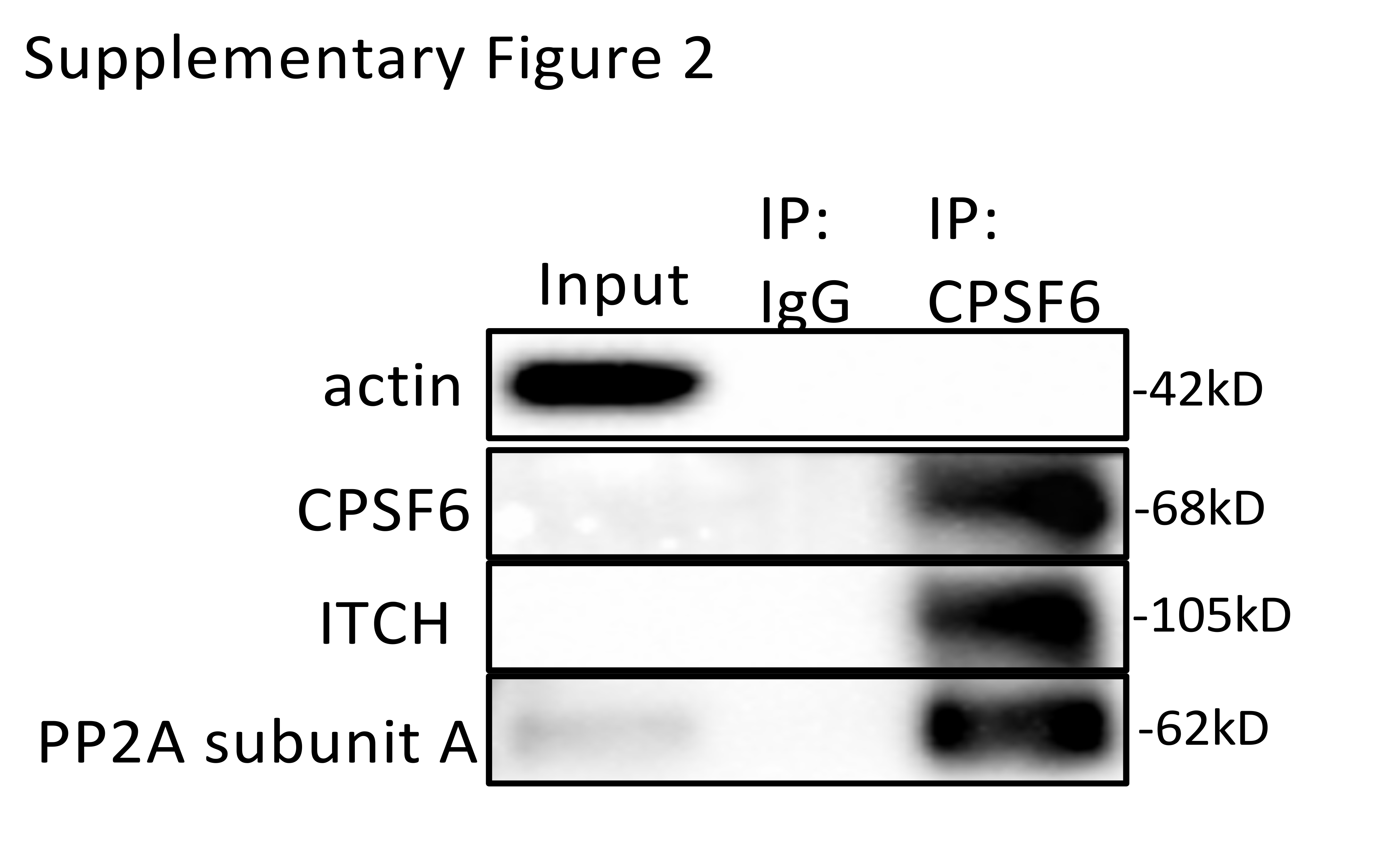

Supplement: FIG S2 [file mbio.01098-21-sf002.tif]
