# Supplementary material for: Cleavage and Polyadenylation Specificity Factor 6 Is Required for Efficient HIV-1 Latency Reversal
Source: mBio. 2021 Jun 22;12(3):e01098-21. doi: 10.1128/mBio.01098-21 (PMC8262898; doi:10.1128/mBio.01098-21)
Supplement: TEXT S1 [file mbio.01098-21-s0001.docx]

**Supplementary Methods 1**

**Virus production**

HEK293FT cells (1.8 x 10^7^) were seeded into a T175 flask one day prior to transfection. Plasmids pNL4.3-ΔEnv-nLuc-2ANef (18 μg) and pCMV-VSVG (7 μg) were prepared in 900 μl ddH_2_O and then mixed with 100 μl 2.5 M CaCl_2_. One ml of HEPES-buffered saline 2X (Sigma, US) was added to the mixture. The DNA-calcium phosphate mixture was then added to cells with fresh culture medium containing 2 μM chloroquine. Cell supernatant was collected after 2 days and filtered with a 0.22 μm filter (VWR, US). Viruses were aliquoted and stored at -80°C.
