# Supplementary material for: Cleavage and Polyadenylation Specificity Factor 6 Is Required for Efficient HIV-1 Latency Reversal
Source: mBio. 2021 Jun 22;12(3):e01098-21. doi: 10.1128/mBio.01098-21 (PMC8262898; doi:10.1128/mBio.01098-21)
Supplement: TABLE S1 [file mbio.01098-21-st001.docx]

**Supplementary Table 1 Sequences of gRNAs**

| **Name** | **Sequence** |
| --- | --- |
| CXCR4 gRNA | GAAGCGTGATGACAAAGAGG |
| CPSF6 gRNA | ATAGACATTTACGCGGATGT |
| NF-kB p65 gRNA | GAGGGGGAACAGTTCTGAAA |
| CPSF5 gRNA | CAGCCGGTCTGCGAGCGATT |
| ITCH gRNA | CCGGCTGCCATCTTAGTCTA |
