# Supplementary material for: Cleavage and Polyadenylation Specificity Factor 6 Is Required for Efficient HIV-1 Latency Reversal
Source: mBio. 2021 Jun 22;12(3):e01098-21. doi: 10.1128/mBio.01098-21 (PMC8262898; doi:10.1128/mBio.01098-21)
Supplement: TEXT S2 [file mbio.01098-21-s0002.docx]

**Supplementary Methods 2**

**CRISPR-Ca9-mediated KO**

Guide RNAs were designed using the web tools of Dr. Zhang’s laboratory (<https://zlab.bio/guide-design-resources>). Guide RNAs (Supplementary Table 1, IDT, US) were mixed with tracrRNA (IDT, US) and heated at 95°C for 5 min. Recombinant *S.pyogenes* Cas9 nuclease (IDT, US) was added to the RNA mixture and kept at room temperature for 20 min. Cells were washed with PBS and resuspended in Buffer R (for SupT1 cells) or Buffer T (for primary CD4+ cells) from the Neon Transfection System 10μl kit (ThermoFisher, US). Pre-assembled Cas9-gRNA RNPs were electroporated into cells using a Neon unit (ThermoFisher, US). Cells were then added into a pre-warmed plate and cultured in a 37°C incubator containing 5% CO_2_ for 2 days. Knockout efficiency was measured via either flow cytometry or western blot.
