# Supplementary material for: Cleavage and Polyadenylation Specificity Factor 6 Is Required for Efficient HIV-1 Latency Reversal
Source: mBio. 2021 Jun 22;12(3):e01098-21. doi: 10.1128/mBio.01098-21 (PMC8262898; doi:10.1128/mBio.01098-21)
Supplement: TEXT S3 [file mbio.01098-21-s0003.docx]

**Supplementary Methods 3**

**Flow cytometry**

For CD4 and P24 staining, 3 x 10^5^ cells from each sample were washed with PBS in 5 ml round bottom flow cytometry tubes (VWR, US), and stained with 0.1 µl Fixable Viability Dye eFluor 450 (eBioscience, US) and 0.1 µl anti-CD4-APC (Invitrogen, US) in 100 µl PBS at 4°C for 30 min. Cells were washed again with PBS and centrifuged at 300 g for 5 min. 100 µl Cytofix/Cytoperm (BD, US) was added to each tube and kept at 4°C for 1 h. Fixed and permeablized cells were washed with Permwash buffer (BD, US) and then incubated with 0.5 µl anti-HIV-1 core (P24)-FITC (Beckman Coulter, US) in 100 µl Permwash buffer at 4°C for 2 h. Cells were washed with PBS and resuspended in 250 µl PBS. All samples were run on BD LSRFortessa^TM^ X-20 (BD, US). Data was analyzed in FlowJo^TM^ 10.7.1 (BD, US).
