# Supplementary material for: Cleavage and Polyadenylation Specificity Factor 6 Is Required for Efficient HIV-1 Latency Reversal
Source: mBio. 2021 Jun 22;12(3):e01098-21. doi: 10.1128/mBio.01098-21 (PMC8262898; doi:10.1128/mBio.01098-21)
Supplement: TABLE S2 [file mbio.01098-21-st002.docx]

**Supplementary Table 2 Sequences of RT-qPCR primers**

| **Name** | **Sequence** |
| --- | --- |
| HIV-1 forward primer | GCTAACTAGGGAACCCACTG |
| HIV-1 reverse primer | GTTACCAGAGTCACACAACAGAC |
| PP2A subunit Aa (PPP2R1A) forward primer | AACTTCGACAGTACTTCCGG |
| PP2A subunit Aa (PPP2R1A) reverse primer | GGCCAGGTTGGAGAACAT |
| PP2A subunit Ab (PPP2R1B) forward primer | ATTTTCAGAGCTCCAGAAAGCC |
| PP2A subunit Ab (PPP2R1B) reverse primer | TCAATGGGCAAGTTCTCACC |
