# Supplementary material for: Cleavage and Polyadenylation Specificity Factor 6 Is Required for Efficient HIV-1 Latency Reversal
Source: mBio. 2021 Jun 22;12(3):e01098-21. doi: 10.1128/mBio.01098-21 (PMC8262898; doi:10.1128/mBio.01098-21)
Supplement: TABLE S3 [file mbio.01098-21-st003.docx]

**Supplementary Table 3 Antibodies for western blot**

| **Antibody name** | **Company** | **Catalog number** |
| --- | --- | --- |
| goat anti-mouse IgG | Jackson Immuno Research | 115-035-146 |
| goat anti-rabbit IgG | Jackson Immuno Research | 111-035-046 |
| mouse anti-human actin | Sigma | A5441 |
| rabbit anti-human CDK9 pThr186 | Cell Signaling Technology | 15102 |
| rabbit anti-human CDK9 | Santa Cruz | 15777 |
| mouse anti-human CPSF5 | Santa Cruz | 81109 |
| rabbit anti-human CPSF6 | Abcam | 175237 |
| mouse anti-human CXCR4 | Santa Cruz | 53534 |
| rabbit anti-human lamin b1 | Cell Signaling Technology | 12856 |
| rabbit anti-human ITCH | Cell Signaling Technology | 12117 |
| rabbit anti-human p65 | Biolegend | 622601 |
| mouse anti-human Pol II | Santa Cruz | 17798 |
| rabbit anti-human Pol II Ser2P | Cell Signaling Technology | 13499 |
| rabbit anti-human Pol II Ser5P | Cell Signaling Technology | 13523 |
| rabbit anti-human PP2A subunit A | Cell Signaling Technology | 2041 |
| rabbit anti-human PP2A subunit B | Cell Signaling Technology | 4953 |
| rabbit anti-human PP2A subunit C | Cell Signaling Technology | 2259 |
