# Supplementary material for: Cleavage and Polyadenylation Specificity Factor 6 Is Required for Efficient HIV-1 Latency Reversal
Source: mBio. 2021 Jun 22;12(3):e01098-21. doi: 10.1128/mBio.01098-21 (PMC8262898; doi:10.1128/mBio.01098-21)
Supplement: TEXT S4 [file mbio.01098-21-s0004.docx]

**Supplementary Methods 4**

**Protein extracts for western blot**

Total protein extract was obtained by lysing cells in NETN buffer (100 mM NaCl, 20 mM Tris-Cl pH 8.0 and 0.5 mM EDTA) containing the phosphatase inhibitor cocktail (Roche, US) and protease inhibitor cocktail (Roche, US) (NETN+/+ buffer). Nuclear protein extract was obtained by lysing cells first in 100 µl cell lysis buffer (5 mM PIPES pH 8.0, 85 mM KCl, 0.5% NP40, phosphatase inhibitor and protease inhibitor cocktails) on ice for 30 min. Lysates were centrifuged at 4°C 900 g for 20 min. Resulting nuclei were washed with cold PBS and resuspended in 100 µl nuclear lysis buffer (50 mM Tris pH 8.1, 10 mM EDTA, 1% SDS, phosphatase inhibitor and protease inhibitor cocktails). The resuspension was boiled for 20 min, and then centrifuged at 16,200 g for 10 min. Supernatants were stored at -80°C.
